# New Insights into the Anti-Aging Mechanism of Collagen Peptides—Emphasis on Lysosomes and Mitochondria Function

**DOI:** 10.3390/molecules31050763

**Published:** 2026-02-25

**Authors:** Wei Huang, Jinshan Ran, Yanli Du, Changwei Cao

**Affiliations:** 1 College of Agronomy and Life Sciences, Kunming University, Kunming 650214, China; huangwei_kmu@163.com (W.H.);; 2 College of Biological and Food Engineering, Southwest Forestry University, Kunming 650224, China

**Keywords:** collagen peptides, skin aging, precise anti-aging, lysosomes, mitochondria

## Abstract

With the intensification of social aging and the improvement of living standards, delaying aging has become a focus of common concern, especially in regard to skin aging. Although collagen peptides have been widely reported as therapeutic agents in relieving skin aging, the molecular mechanisms remain inadequately elucidated. This review emphasizes that the alleviation of skin aging by collagen peptides is a systematic and complex process, including the removal of reactive oxygen species, inhibition of inflammation, inhibition of extracellular matrix (ECM) degradation and melanin deposition, activation of lysosomal and mitochondrial function, and promotion of ECM synthesis. It also highlights that lysosomes and mitochondria may be the key organelles that regulate collagen peptides to alleviate skin aging. Current research on the mechanism of collagen peptides in alleviating skin aging still requires bold breakthroughs and should not be confined to the transforming growth factor (TGF-β)/Smad, mitogen-activated protein kinase, and nuclear factor kappa-B pathways. In addition, many natural antioxidant components have been proven to alleviate skin aging by regulating organelle function. Therefore, the regulatory effects of collagen peptides with antioxidant activity on mitochondrial and lysosome functions in aging skin need more attention and exploration, which is of great significance for further research on precise skin care and targeted anti-skin aging therapy.

## 1. Introduction

Whether we can stop our aging is a scientific question that has puzzled humanity for thousands of years. Aging is a gradual and comprehensive degradation of physiological functions and has always been considered inevitable. However, with the deepening of anti-aging research, preventing or mitigating aging is gradually being considered feasible. Dietary interventions, nutrition, and metabolic regulation have profound effects on skin health and even longevity. Skin is the body’s barrier against external stimuli and direct reflects the body’s health and beauty. With population aging, declining birth rates, improved living standards, and the outbreak of COVID-19, people are paying more attention to managing skin health. They are beginning to realize the effectiveness of disease prevention and health management through diet management or the supplementation of foodborne functional ingredients. Moreover, delaying aging through diet intervention is a safe, broad-spectrum, and low-cost strategy [1,2]. Therefore, clarifying the functions and mechanisms of food and developing safe and effective functional food have become common goals of researchers and consumers. Supplementing functional active peptides to improve the condition of aging skin has become a research topic of interest, with collagen peptides in particular being used as therapeutic agents for skin repair.

Collagen is a cylindrical biopolymer protein and the main component of the animal extracellular matrix (ECM). Collagen peptides are short peptide chains derived from the hydrolysis of collagen and consist of 2 to 20 amino acid residues. Due to their excellent functional activity, they play a significant role in the health management of skin, joints, muscles, and the cardiovascular system [3]. Although collagen peptides have long been used as therapeutic agents for skin repair, increasing studies have also confirmed that collagen peptide supplementation can improve the condition of aging skin [4,5], but the molecular mechanism of collagen peptides in alleviating skin aging is still not completely clear. In addition, with the concepts of precise nutrition, precise skin care, and targeted therapy gaining more and more attention, it is necessary to re-discuss the mechanism of collagen peptides in alleviating skin aging.

This review searched three databases—Web of Science, Google Scholar, and CNKI—for relevant literature over the past decade on ‘collagen peptides’, ‘skin aging’, and ‘mechanisms,’ excluding unrelated studies. After briefly introducing the characteristics of skin aging and the molecular mechanism leading to skin aging, this study reviews the research literature on the molecular mechanisms of collagen peptide supplementation in alleviating skin aging over the past ten years and identifies the shortcomings of current research and directions for future research. This review will deepen our understanding of collagen peptides in relieving skin aging and provide some strategies and directions for subsequent research.

## 2. Characteristics and Mechanisms of Skin Aging

**Figure 1:**
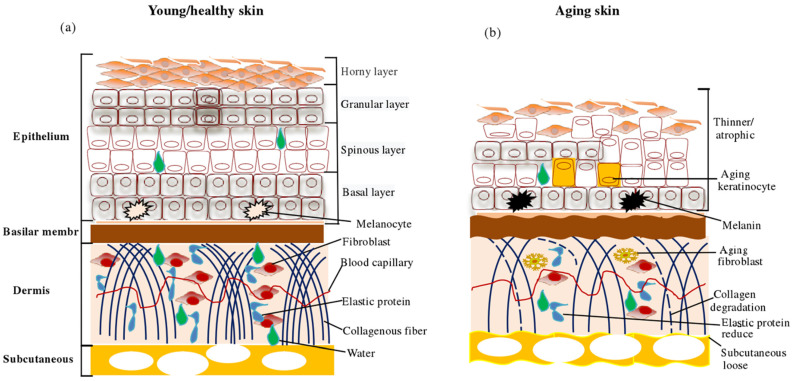
Schematic diagram of skin structure and changes in aging skin structure: (**a**) young/healthy skin; (**b**) aging skin [8].

The skin is the main organ of the body that resists environmental stress, and it is composed of the epidermis, dermis, and subcutaneous tissue (Figure 1). The epidermis is the first barrier against the invasion of bacteria and viruses. The dermis is connective tissue composed of fibroblasts, responsible for synthesizing and secreting collagen and matrix proteins for the extracellular environment, which gives skin elasticity and strength. The subcutaneous tissue is the fat layer below the dermis, which plays the role of connecting skin, muscle, and bone, regulating the balance of the internal skin environment, and so on [6].

Skin aging is a phenomenon in which the skin’s ability to adapt to the environment decreases, and cells gradually tend to die. It is caused by both internal and external factors and can be divided into chronic aging and photo-aging. The main characteristics are the accumulation of intracellular macromolecular damage, impaired ability of stem cells to promote tissue renewal, and progressive loss of skin physiological integrity. Histological analysis showed epidermal atrophy, a reduced number of dermal fibroblasts and collagen fibers, and tissue that is loose, sparse, or even absent. This is caused by a reduced ability of keratinocyte stem cells to renew and repair the skin; the accumulation of skin fibroblast injury and dysfunction, loss of the ability to reshape the tissue extracellular matrix, and disruption of skin cell homeostasis [7]. Aging skin appears wrinkly, slack, and rough, and the color is yellowish or grayish yellow, with telangiectasia or pigment spot formation. The main molecular mechanisms leading to skin aging are cellular oxidative stress, DNA damage, gene mutation, telomere shortening, microRNA expression, accumulation of advanced glycosylation end products, and skin inflammation [8]. As stem cell transplantation, hormone therapy, telomerase modification, retinoic acid, and other treatments for skin aging have their own disadvantages, safe and abundant collagen peptides have become a hot research topic.

## 3. Absorption and Bioavailability of Collagen Peptides

Evaluating the absorptivity, bioavailability, or the amount of collagen peptides that reach specific target tissues is a fundamental requirement for discussing the anti-aging effects of collagen peptides. However, these aspects are among the most lacking and challenging areas in current research reports. Since the digestion and absorption of collagen peptides in the gastrointestinal tract is a complex process, it is difficult to evaluate its absorption efficiency, absorption form, bioavailability, and the extent to which they reach the target tissue in vivo. Wang et al. [9] demonstrated that after oral administration of collagen (4000 mg/kg bw), the bioavailability of collagen in rats was approximately 50%, and more than 63.4% was absorbed into the intestine in the form of peptides. Collagen can be directly and effectively absorbed into plasma and quickly transferred into skin, bone, joint and other tissues when administered in the form of tripeptides. The plasma dynamics of oral and abdominal administration are similar [10]. Similarly, Yazaki et al. [11] also proved that functional peptides can be transferred to the skin after dietary supplementation of collagen peptides (oral, 300 mg/kg bw), with Gly-Pro-Hyp being the predominant peptide in plasma and Pro-Hyp being predominant in skin. More convincingly, in one study, after rats were fed radio-labeled (C^14^) collagen peptides, the plasma radioactivity level of rats increased rapidly, and collagen peptides were absorbed into the blood in the form of tripeptides, which remained in the kidney, skin, and other tissues. Even after 14 days of administration, the rat skin still maintained a high radioactivity level [12].

However, although previous studies have shown that oral supplementation of collagen peptides is digested and absorbed into the skin, these studies have not been systematic or complete. In brief, studies on the anti-aging effects of collagen peptides lack evaluation of the digestion, absorption, and utilization of collagen peptides, while research on collagen bioavailability lacks evaluation of its effect against skin aging. Moreover, it is necessary to explore the relationship between molecular weight, electric charge, and hydrophobicity of collagen peptides and their digestive stability, intestinal transport, and bioavailability for a systematic evaluation of the effect of collagen peptides on alleviating skin aging [13].

## 4. Mechanisms of Collagen Peptides in Anti-Skin Aging

### 4.1. Scavenging Free Radicals, Inhibiting Inflammation

**Figure 2:**
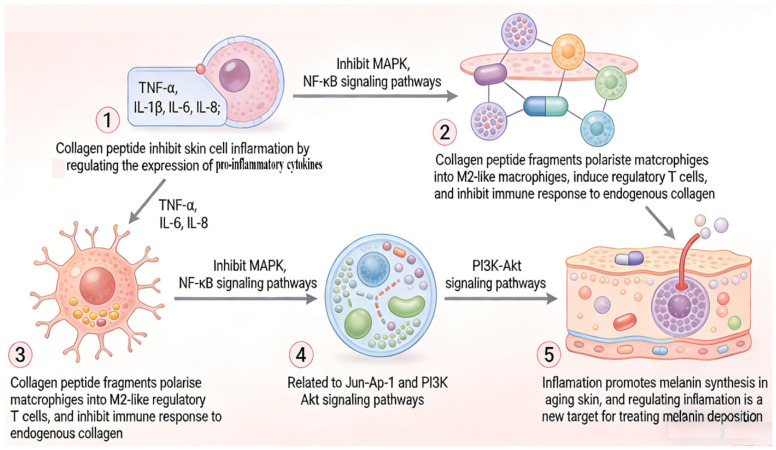
Mechanism of collagen peptides in alleviating skin aging by inhibiting inflammation. This figure was created using ScienceSlides 16 software. The same applies below.

The main reason for skin aging is that reactive oxygen species (ROS) accumulated by oxidative stress in aging skin exceed the body’s antioxidant defense ability, which destroys the ECM and causes inflammation. Mechanistically, excessive ROS activate the nuclear factor kappa-B (NF-κB) and mitogen-activated protein kinase (MAPK) signaling pathways, activating or promoting the expression of activator protein 1 (AP-1) and NF-κB to inhibit the synthesis of skin collagen. This then increases the expression of tumor necrosis factor-α (TNF-α), interleukin (IL)-1α, and matrix metalloproteinases (MMPs), inducing ECM degradation and skin inflammation, and thereby accelerating skin aging [2,14]. It has been widely reported that supplementation with collagen peptides possessing antioxidant activity can increase antioxidant enzyme activity in aging skin [15,16]. Regarding anti-inflammatory activity, many studies have reported that collagen peptides have an anti-inflammatory effect on aging skin [17]. Mechanistically (Figure 2), collagen peptides inhibit skin cell inflammation and improve immune function by down-regulating the expression of TNF-α, IL-1β, IL-6, IL-8, and other pro-inflammatory cytokines, which may be related to the inhibition of MAPK, NF-κB, and other signaling pathways [18,19]. Due to the important role of M2 macrophages in inflammation, collagen peptide fragments can induce regulatory T cells to improve skin health parameters by polarizing macrophages towards M2-like macrophages and inhibiting the immune response to endogenous collagen [20]. In addition, collagen peptides’ inhibition of inflammation may also be related to the Jun/AP-1 and PI3K-Akt signaling pathways [21,22]. Moreover, since inflammation promotes melanin synthesis in aging skin, regulation of inflammation may be a new approach and a new target for the treatment of melanin deposition in aging skin [23].

### 4.2. Activate Lysosomal Function

An important feature of cellular senescence is the accumulation of damaged organelles and protein aggregates. Senescent cells continue to damage the body and inhibit tissue regeneration, so clearing senescent cells or reducing their secretion of inflammatory cytokines will significantly improve the aging phenotype [24]. Lysosomes usually play an important role in the degradation of damaged organelles and protein aggregates in senescent cells [25]. Previous studies have shown that cell life depends on the function of lysosomes. Lysosomes are cell centers and signal transduction centers that regulate cell and body homeostasis, control aging and lifespan, sense the nutritional and energy status in cells, and regulate cell growth and metabolism through the regulatory factor mammalian target of rapamycin (mTOR) located on the surface of lysosomes [26,27,28]. Damaged lysosomes are a marker of aging and age-related diseases. In addition to degrading biomolecules and removing damaged cell components, lysosomes also play an important role in nutrition perception and cellular immunity [29]. During aging, activated lysosomes not only remove aggregates but also enhance the viability of aging neural stem cells [30]. Moreover, activation of lysosomal function also reduces intracellular ROS concentration and prevents cell dormancy. Similarly, any functional decline of lysosomes will increase intracellular ROS concentration and ultimately promote cell dormancy [31]. Autophagy and lysosomal degradation play a protective role for cells under cellular stress or nutrient deprivation. However, UVA impairs this process by damaging lysosomal function in dermal fibroblasts, thereby inhibiting autophagy and lysosomal degradation [32]; loss of collagen VII in keratinocytes results in lysosomal homeostasis disturbances [33]. Collagen peptides have been proven to remodel the extracellular matrix of aging skin. Miao et al. [34] reported that lysosomal function in the epidermal cells of nematodes during molting (ECM renewal) was specifically activated, which promoted the degradation of old cell components and the utilization of degradation metabolites for resynthesis. Walnut protein peptide (50 μM) promotes autophagy and lysosomal-related protein expression through the Akt/mTOR signaling pathway to relieve cellular oxidative stress [35]. Similarly, a previous study found that the lysosome pathway was significantly enriched by upregulated genes in mouse aging skin after supplementation with chicken bone collagen peptides (oral, 200–1000 mg/kg bw) [21]. More similar studies are summarized in Table 1. Therefore, lysosomal function may play an important role in the process of collagen peptides relieving skin aging, but further verification is needed.

### 4.3. Activate Mitochondrial Function

Mitochondria are the energy and metabolic centers of eukaryotic cells and play a major role in energy production and oxidative stress. They are the primary organelles affected during skin aging, with mitochondrial dysfunction being the main manifestation. Mitochondria are the main source of ROS in cells and serve as a regulatory hub for cellular and biological signaling pathways [46,47]. It has been confirmed that oxidative damage caused by excessive ROS production in mitochondria is the molecular basis for many pathophysiological conditions, including aging [48]. Mitochondrial dysfunction and oxidative stress are key features of aging tissues, including skin aging, and are directly related to skin aging phenotypes [49]. As an ECM protein, VI collagen deficiency leads to mitochondrial dysfunction and apoptosis in mice [50]. Son and Lee [51] even point out that all pathways associated with aging and age-related diseases point to mitochondria. For example, dermal fibroblasts irradiated by UVA exhibit decreased mitochondrial function, resulting in insufficient type I collagen and fibrillin-1 fiber formation [52]. Increasing evidence shows that mitochondrial function plays an important role in skin health, aging, and age-related disease [48,53]. Mitochondrial dysfunction drives skin aging, so using mitochondria-targeted antioxidants to reduce mitochondrial oxidative stress is a promising strategy to prevent and treat skin damage [48,53].

However, restoring mitochondrial function can reverse skin wrinkling and hair loss in mice [53]. Pyruvate supplementation protects skin fibroblasts from aging by regulating the mitochondrial and lysosome functions of dermal fibroblasts and increasing the production of oxidized nicotinamide adenine dinucleotide (NAD^+^) [54]. Natural mitochondrial-targeting drugs with antioxidant properties are effective methods to prevent and treat UV-induced skin damage by reducing mitochondrial oxidative stress [55]. Mitochondria are the primary sites where the Gly-Pro-Ala peptide, isolated from fish skin gelatin hydrolysate, alleviates inflammation and oxidative stress in intestinal epithelial cells (mice oral, 100 mg/kg bw) [56]. Two antioxidant active peptides (TGIIT and YAR), isolated from milk fat globular membrane protein peptides obtained from in vitro digestion, can regulate mitochondrial functional activity by reducing mitochondrial vacuolization and autophagy, thereby alleviating cellular oxidative stress [44]. Bhullar et al. [57] reported that supplementation of elastin peptides in vivo can increase the generation of nitric oxide synthase mRNA, suggesting that these peptides can regulate mitochondrial function in mice, and emphasized that active peptides derived from food have the ability to improve mitochondrial function to alleviate aging. A novel peptide (mice oral, 100 mg/kg bw) from the hippocampus has been shown to improve mitochondrial function and protect muscle fibers through the AMPK/PGC-1α signaling pathway [58]. Polypeptides derived from *Chlamys farreri* can alleviate mitochondrial damage of human skin fibroblasts (cell, 100 μg/mL) induced by UVB, improve and maintain mitochondrial transmembrane potential, and their protective effect is positively correlated with peptide concentration [59,60]. Other studies found that after oral supplementation of chicken bone collagen peptides, differential metabolites in the aging skin of mice were significantly enriched in metabolic pathways, including pyruvate metabolism and tricarboxylic acid metabolism, all of which point to mitochondria, suggesting that collagen peptides may promote these metabolic pathways to alleviate skin aging by activating mitochondrial function [21]. Tilapia skin peptides showed improvement in dysfunctional mitochondria in mice (oral, 3 g/kg bw) [61]. Mitochondrial membrane potential is an important parameter reflecting mitochondrial function, which rapidly declines with mitochondrial dysfunction; preventing the decline of mitochondrial membrane potential could slow down the rate of aging. Treatment with a synthetic peptide (Gal2-Pep) with excellent antioxidant activity can significantly improve the mitochondrial membrane potential of aging skin fibroblasts (100 mM), activate cell mitochondria, and show strong anti-aging activity [62,63]. It has also been reported that mitochondrial transfer techniques mediated by the cell-penetrating peptide Pep-1 can restore mitochondrial function and serve as a potential therapeutic intervention for mitochondrial diseases [64].

**Figure 3:**
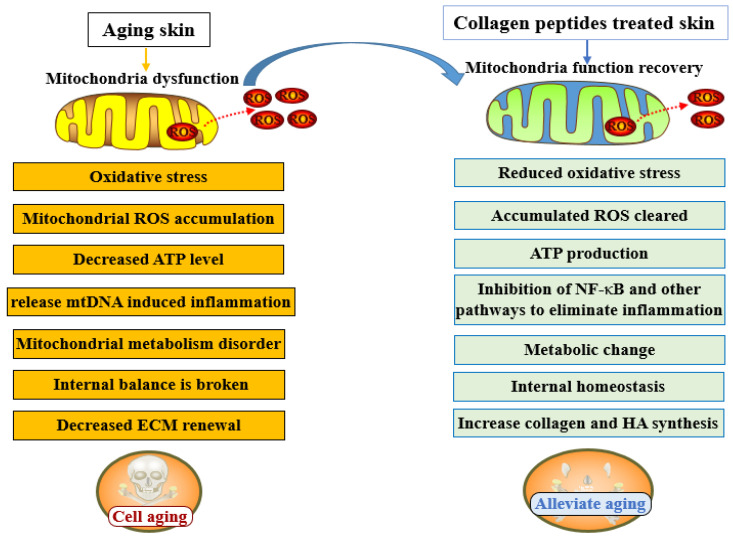
Mitochondrial dysfunction and the effect of collagen peptide treatment on mitochondrial function. In the figure, ROS = reactive oxygen species; NF-κB = nuclear factor kappa-B; ECM = extracellular matrix; HA = hyaluronic acid; ATP = adenosine triphosphate. The same applies below.

In addition, when certain mitochondrial components and metabolites are released into the cytosol or extracellular environment, they promote inflammatory response and destroy the immune system. Moreover, damaged mitochondria release mitochondrial DNA and activate multiple inflammatory pathways, including the NF-κB signaling pathway. Therefore, mitochondria are also the main regulatory factors of inflammation and may be an effective means to control inflammation by regulating mitochondrial function [65,66,67]. Inflammation is one of the main causes of skin aging, and numerous studies have shown that supplementary collagen peptides inhibit the skin inflammatory response [68]. Fish collagen oligopeptides (25, 50, 100 µg/mL) can promote fibroblast homeostasis, inhibit inflammation, and protect mitochondria; their mitochondrial protective molecular mechanism may involve the NAD^+^/SIRT1/PGC1α signaling pathway [69]. Therefore, collagen peptides may also inhibit inflammatory responses in aging skin by regulating mitochondrial function. Similarly, as the energy and metabolic center of cells, the integrity of mitochondrial function is crucial for collagen peptides to promote the synthesis of collagen, hyaluronic acid, and other extracellular substrates in aging skin. Therefore, collagen peptides may also promote the synthesis of ECM in aging skin by activating mitochondrial function to alleviate skin aging. With the advancement of the concept of precise skin care and mitochondria being regarded as an important target of skin care, especially in the field of anti-aging and repair, it is believed that mitochondria will become a hot spot of anti-aging research [70] (Figure 3).

### 4.4. Inhibition of Skin Moisture Loss and Melanin Production

**Figure 4:**
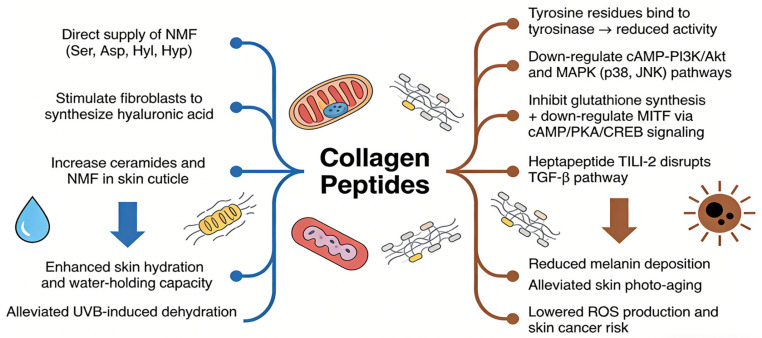
Pathways of collagen peptides inhibit skin water loss and melanin production.

Moisture loss and melanin deposition are important features of aging skin. The reduction of natural moisturizing factors such as urea, uric acid, and hyaluronic acid in aging skin is the main reason for the reduction of skin hydration capacity and water content (Figure 4). A large number of studies have shown that collagen peptides supplementation can improve skin moisture content, which may be due to the fact that collagen peptides contain a large number of natural moisturizing factors (NMFs), such as Ser, Asp, Hyl, Hyp, and hydrophilic radicals (hydroxyl and carboxyl radicals), which enhance skin hydration and water-holding capacity [71,72]. For example, oral supplementation of collagen peptides (500 and 1000 mg/kg bw) derived from tilapia scales alleviated UVB-induced skin dehydration and water loss in mice by regulating hyaluronic acid synthesis [73]. Mechanistically, collagen peptides stimulate fibroblasts to synthesize hyaluronic acid, which is known as the “reservoir” of skin because it contains a large number of hydrophilic radicals [21,74]. Since ceramides and NMFs play an important role in maintaining skin moisture, ingestion of collagen peptides derived from *Nemipterus virgatus* scales (1000 mg) also increased volunteers’ skin moisture content by increasing ceramides and NMF levels in the skin cuticle [75].

Melanin is a kind of biopolymer, which is synthesized by melanocytes under the induction of ultraviolet radiation and other factors and is closely related to skin photo-aging. An appropriate amount of melanin can reduce the direct absorption of nucleic acid to ultraviolet radiation and avoid skin lesions induced by nuclear DNA damage. However, melanin also damages the skin by producing ROS, and excess melanin will cause diseases such as skin cancer [76]. Tyrosinase is a key enzyme that converts tyrosine into melanin. Melanin is synthesized by multiple genes, molecules, and pathways regulation, including cycladenosine monophosphate (cAMP), MAPK, and TGF-β signaling pathways. It has been reported that collagen peptides with the potential to inhibit skin melanin production have been found in animal tissues from various sources [77]. The reason is that tyrosine residues in collagen peptides can bind with tyrosinase to reduce the activity of tyrosinase and inhibit the production of melanin in skin, and the ability of collagen peptides to inhibit the activity of skin tyrosinase is affected by the source, composition, and molecular weight of the peptides [74,78]. Collagen peptides inhibit melanin production in the following ways (Figure 4): (1) decreasing melanin production in melanoma cells (B16F1) by down-regulating the cAMP-PI3K/Akt and MAPK pathways (p38 and JNK) [79]; (2) inhibiting melanogenesis by improving glutathione activity to remove free radicals, inhibit tyrosinase activity, and block melanin synthesis, and down-regulating the expression of melanocyte-induced transcription factors (by inhibiting cAMP/protein kinase A/cAMP response binding protein signaling and nitric oxide production) [79]. In addition, the N-terminal cysteine-containing heptapeptide TILI-2 may inhibit melanin production by disrupting the TGF-β signaling pathway in B16F1 cells [80]. However, the exact mechanism by which collagen peptides inhibit tyrosinase activity and thus reduce melanin content has not been fully elucidated.

### 4.5. Regulation of Skin ECM Degradation and Synthesis

**Figure 5:**
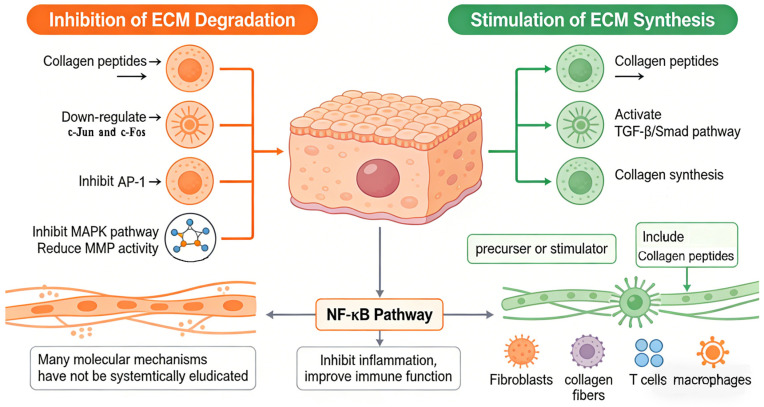
Pathways by which collagen peptides inhibit skin water loss and melanin production.

Collagen peptides regulate the degradation and synthesis of ECM (mainly collagen and hyaluronic acid) in aging skin, which is the most studied and widely reported pathway for collagen peptides to alleviate skin aging. Mechanistically (Figure 5), collagen peptides can directly act on skin fibroblasts after digestion and absorption. On the one hand, collagen peptides inhibit the activation of AP-1 by down-regulating the expression of transcription factors c-Jun and c-Fos that regulate the expression of MMPs, leading to the blocking of the MAPK pathway and then inhibiting the degradation of ECM (mainly inhibiting the degradation of collagen fibers). Molecular docking tests also found that collagen peptides may reduce the activity of MMPs and inhibit ECM degradation by forming active sites with MMPs [81,82]. On the other hand, collagen peptides participate in the synthesis of matrix collagen by activating the TGF-β/Smad pathway to alleviate skin aging, including collagen peptides as a precursor or stimulator of collagen synthesis [74,83]. In addition, collagen peptides regulate ECM degradation and synthesis to alleviate aging, which may also be related to blocking the NF-κB pathway, inhibiting inflammation, and improving immune function. Although previous studies have confirmed that collagen peptides alleviate skin aging through the NF-κB, MAPK, and TGF-β/Smad pathways, most of the research seems to have only repeatedly verified these pathways, lacking new discoveries and explorations, such as the role of T cells and macrophages, which are the body’s first immune response to ingested collagen peptides. This is because the biological process by which collagen peptides alleviate skin aging is complex, and many molecular mechanisms have not been systematically elucidated.

## 5. Inducing Immune Cells to Increase Skin Turnover

The key mechanism by which collagen peptides prevent skin aging is reshaping the skin ECM. In this process, properly removing and managing aging ECM components is a critical issue. It has been confirmed that macrophages play a key role in cell uptake, ECM turnover and remodeling during collagen transformation [84,85]. As key regulators of the immune system, macrophages can sense any input from the body’s microenvironment and transform these inputs into different responses. Tregs can induce macrophages to differentiate into M1 and M2 regulatory phenotypes, leading to different functional performances. For example, M1-like macrophages promote the removal of dead cells, while M2-like macrophages play an important role in eliminating inflammation and promoting tissue remodeling [86]. Meisam et al. [20] reported that oral ingestion of collagen peptides alleviates skin aging and improves skin conditions by inducing Tregs and M2-like macrophages to increase skin turnover, including oral tolerance and non-tolerance mediated mechanisms. In brief, supplemented collagen peptides mediate Tregs to induce macrophages to differentiate into the M2 phenotype, thereby eliminating tissue inflammation and promoting tissue renewal. This is a new perspective, but the current research literature is limited, and more studies are needed to support this view.

## 6. Conclusions and Prospects

**Figure 6:**
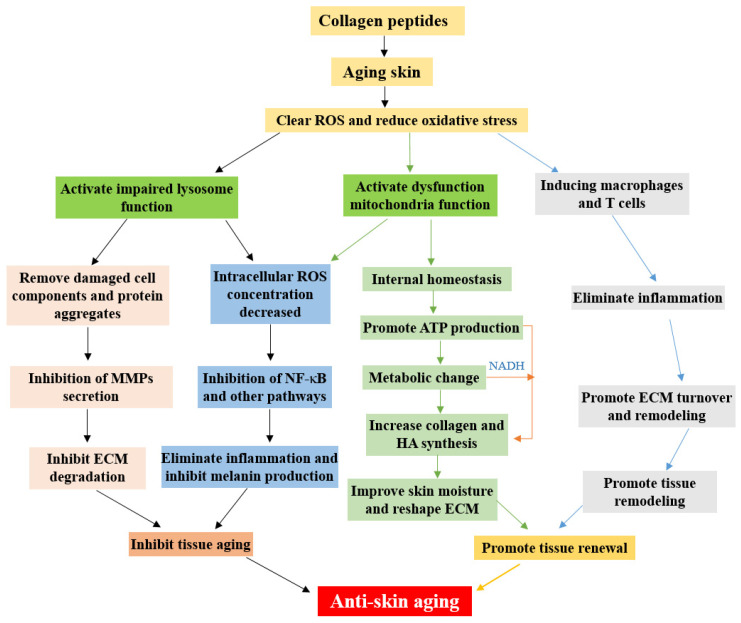
Potential biological process of supplemented collagen peptides against skin aging. MMPs = matrix metalloproteinases.

Skin aging is a biological process affected by multiple factors, and collagen peptides have been used as a therapeutic agent to improve skin conditions. The existing literature consistently confirms the effectiveness of collagen peptide supplementation in enhancing skin health, and it is widely accepted that collagen peptides alleviate skin aging through pathways such as NF-κB, MAPK, and TGF-β/Smad. However, many aspects remain to be further elucidated or validated. For instance, there is limited research on the absorption and utilization efficiency of supplemented collagen peptides and their clinical evaluation. Additionally, there is lack of studies exploring the interactions between collagen peptides and other food nutrients during digestion. Furthermore, there is no unified standard for the specific dosage, form, or method of collagen peptide supplementation. Mechanistically, most studies are confined to the aforementioned pathways, merely repeating validations, and lack new breakthroughs or attempts. Based on an analysis of the existing literature, we propose that collagen peptides with antioxidant activity may first remove ROS, inhibit inflammation and ECM degradation, then activate lysosomal function to remove damaged organelles and accumulated protein aggregates, further reducing ROS. Finally, they activate mitochondrial function to promote a series of biochemical reactions and serve as a precursor in the biosynthesis of collagen fibers, hyaluronic acid, and other components to alleviate skin aging. In addition, mitochondria and lysosomes may be the targeted organelles and centers through which collagen peptides alleviate skin aging (Figure 6). Although there are few studies on anti-aging and skin repair with mitochondria or lysosomes as targets, with the advancement of precise skincare and anti-aging concepts, we believe that mitochondria and lysosomes will become a new focal point in the field of precise anti-aging.

Future research is needed to clarify the following issues regarding mitochondria and lysosomes: (1) whether collagen peptides really alleviate skin aging by regulating mitochondrial and lysosomal functions; (2) how collagen peptides regulate skin aging through mitochondria and lysosomes; (3) whether mitochondrial and lysosomal dysfunction are the main or secondary causes of skin aging; (4) can collagen peptides (or other antioxidant peptides) targeting mitochondria or lysosomes slow down skin aging?

## Figures and Tables

**Table 1:** Summary of research on peptide-regulated mitochondrial or lysosomal functions in alleviating skin aging.

References/Study Type/Country	Source	Treatment Method/Dosage	Main Results	Main Conclusion
[36]/Cell/Britain	Sea cucumber extract mixed peptides (SCMPs)	0.01, 0.1, 0.5, 1 mg/mL, 48 h	SCMPs can promote cell migration and protect organelles such as mitochondria and the endoplasmic reticulum (ER) by regulating the expression of proteins related to the ribosome pathway, glycolysis/glycogenesis, and protein processing in the ER, thereby alleviating the stress response.	SCMP has a strong antioxidant effect and great potential for alleviating oxidative DNA damage and mitochondrial degradation.
[37]/Cell/China	Collagen I	1 mg/mL, 4 h	Molecular collagen I can restore mitochondrial dysfunction caused by the loss of PINK1/parkin-mediated mitochondrial autophagy in HaCaT cells treated with UVB, thereby resisting UVB damage.	Collagen I helps cells recover after UVB radiation by promoting mitochondrial autophagy.
[38]/Cell/China	Radiation-induced frog skin peptide-2 (RIFSP-2)	15 μM	RIFSP-2 enhances the energy production capacity of irradiated skin cells, reduces the accumulation levels of the mitochondria and total reactive oxygen species, and inhibits the decline of mitochondrial membrane potential.	RIFSP-2 protects the mitochondrial function of skin cells exposed to radiation.
[39]/Cell/Korea	Starfish-derived extracts (SDEs)	10 μg/mL, 24 h	SDE inhibited the activity of senescence-associated β-galactosidase and pro-inflammatory cytokines. It promoted mitochondrial autophagy, reduced reactive oxygen species accumulation, and improved mitochondrial function.	SDE promotes mitochondrial autophagy through a PINK1- dependent mechanism and exhibits significant anti-aging effects.
[21]/Mice/China	Chicken bone collagen peptides (CPs)	Oral, 200, 500, 100 mg·kg^−1^, 49 d	Collagen peptides reduce the oxidative level of the skin, inhibit the expression of AP-1 (c-Jun and c-Fos), activate the TGF-β/Smad signaling pathway to promote collagen synthesis, inhibit the expression of MMP-1/3 and reduce skin inflammation to alleviate skin aging in mice.	Lysosomes may be the key pathway for collagen peptides to combat skin aging, and CPS can be used as a functional anti-aging nutritional component.
[40]/Mice/China	Odorrana margaretae skin peptide (OM-GL15)	Oral, 10 nM, 100 nM, 1 mM, 24 h	OM-GL15 inhibits the expression of the p53 protein by suppressing DNA damage in epidermal cells, further inhibiting the mitochondrial apoptotic pathway mediated by caspase-9 and caspase-3, and resisting acute skin injuries.	OM-GL15 has potential value as a drug for preventing UVB-induced skin damage.
[41]/Cell and mice/China	Frog peptide	100 μL, 200 μg/mL, 12 weeks of mice	Frog peptide can alleviate skin photoaging and reduce reactive oxygen species (ROS) levels in mitochondria, but the mechanism by which it reduces ROS remains unclear.	Frog peptides can be regarded as effective antioxidant drugs.
[42]/Cell and mice/China	Hyperoside (HY)	100 μL, 10, 20, 40 mg/mL	HY mediates the interaction between miR-361-5p and the PI3K/AKT/mTOR signaling pathway to maintain mitochondrial dynamic stability, alleviate mitochondrial dysfunction, and enhance mitochondrial autophagy.	HY can significantly improve skin aging, and mitochondria are the key targets.
[43]/Cell/China	Antioxidant peptide ETT	0, 10, 20, 50, 100, 150, 200 μM/2 h	ETT exerts anti-photoaging effects by reducing ROS levels, promoting autophagy, enhancing mitochondrial membrane potential, and inhibiting HaCaT cell apoptosis.	ETT may combat skin aging by maintaining the homeostasis of keratinocytes and reducing cell apoptosis.
[44]/Cell/China	Milk fat globule membrane peptide (MFGMP)	100, 200, 300 μg/mL, 1/2/3/48 h	MFGMP has a good antioxidant effect and protects L6 cells by enhancing mitochondrial function and biosynthesis.	MFGMP offers new insights into antioxidant mechanism research.
[45]/Cell/China	Sheep skin collagen peptide exerts (SSCPs)	0, 0.065, 0.128, 0.773 g/kg/d, 4 weeks	SSCPs alleviate mitochondrial DNA oxidative damage and eliminate ROS. Moreover, they promote mitochondrial biogenesis and energy metabolism through the AMPK/PGC-1α axis.	SSCPs can alleviate mitochondrial dysfunction caused by oxidative stress and improve energy metabolism.

## Data Availability

No new data were created or analyzed in this study. Data sharing is not applicable to this article.
